# 3-D Bioprinting of Neural Tissue for Applications in Cell Therapy and Drug Screening

**DOI:** 10.3389/fbioe.2017.00069

**Published:** 2017-11-17

**Authors:** Michaela Thomas, Stephanie M. Willerth

**Affiliations:** ^1^Department of Mechanical Engineering, University of Victoria, Victoria, BC, Canada; ^2^Division of Medical Sciences, University of Victoria, Victoria, BC, Canada; ^3^Centre for Biomedical Research, University of Victoria, Victoria, BC, Canada; ^4^International Collaboration on Repair Discoveries (ICORD), Vancouver, BC, Canada

**Keywords:** neural tissue engineering, 3-D bioprinting, biomaterials, stem cells, neurodegenerative diseases, drug discovery

## Abstract

Neurodegenerative diseases affect millions of individuals in North America and cost the health-care industry billions of dollars for treatment. Current treatment options for degenerative diseases focus on physical rehabilitation or drug therapies, which temporarily mask the effects of cell damage, but quickly lose their efficacy. Cell therapies for the central nervous system remain an untapped market due to the complexity involved in growing neural tissues, controlling their differentiation, and protecting them from the hostile environment they meet upon implantation. Designing tissue constructs for the discovery of better drug treatments are also limited due to the resolution needed for an accurate cellular representation of the brain, in addition to being expensive and difficult to translate to biocompatible materials. 3-D printing offers a streamlined solution for engineering brain tissue for drug discovery or, in the future, for implantation. New microfluidic and bioplotting devices offer increased resolution, little impact on cell viability and have been tested with several bioink materials including fibrin, collagen, hyaluronic acid, poly(caprolactone), and poly(ethylene glycol). This review details current efforts at bioprinting neural tissue and highlights promising avenues for future work.

## Introduction

Neurodegenerative diseases affect over 55 million individuals annually in North America, creating a multi-billion dollar burden on the health-care industry due to the costs associated with treatment, and rehabilitation therapy (Institute for Neurodegenerative Diseases, 2017). Often selective cell loss in the central nervous system (CNS) leads to these neurodegenerative diseases. Cell therapy can potentially treat neurodegenerative disease by replacing damaged tissues or augmenting remaining cell function (Levy et al., 2016). The basis of cell therapy is that living human cells can be injected into a damaged region of the body to instigate healing (Dove, 2002). Neurodegenerative diseases, such as Alzheimer’s disease, Parkinson’s disease, Huntington’s disease, multiple sclerosis, and amyotrophic lateral sclerosis, as well as neurodegenerative disorders, such as traumatic brain injury, serve as potential candidates for cell therapy as they result in neuronal death in targeted areas of the brain (Vila and Przedborksi, 2003). Neuronal cells possess a low regenerative capacity as they do not proliferate after maturation (Tam et al., 2014). Thus, cell therapy can replace damaged neuronal and support cells, or work indirectly by secreting soluble factors to facilitate the repair process (Tsintou et al., 2015).

While current treatments for these diseases mainly focus on alleviating symptoms and physical rehabilitation, cell therapy can potentially promote cellular repair and remodeling, resulting in improved function. Several issues must be addressed before cell therapy can be widely implemented. These issues include ensuring that the proper number and type of cell are being generated, especially when using stem cells as they can become multiple types of cells. Large quantities of cells are often required for cell therapies to treat neurodegenerative disorders and thus, high-throughput methods for generating these cells must be developed (Rossi and Cattaneo, 2002; Cooke et al., 2010). Direct transplantation of cells in the damaged CNS is possible, but often these cells fail to properly integrate into the brain (Rossi and Cattaneo, 2002). Bioprinting, the use of 3-D printing technology with biocompatible materials that can be seeded with living cells to create tissue constructs, can potentially produce carefully controlled human neural tissue in a consistent rapid manner. The biomaterial scaffolds used in the 3-D printing process are often referred to as bioinks (Skardal and Atala, 2015). Engineered biomaterial microenvironments can help overcome low cell survival rates after transplantation in the damaged CNS and limit migration of cells away from the implantation site while providing a controlled environment for cell growth and differentiation (Cooke et al., 2010; Struzyna et al., 2014). These printable cell scaffolds degrade as the cells develop, either through hydrolysis, or through enzymatic degradation by byproduct proteases, leaving a biologically accurate tissue construct as the result (Freed et al., 1994).

Different types of stem cells have been evaluated *in vitro* and *in vivo* for neural regeneration. These cells include human embryonic stem cells (hESCs), which are pluripotent stem cells derived from a human embryo; mesenchymal stem cells (MSCs), which are multipotent stromal cells that can differentiate into osteoblasts, chondrocytes, myocytes, and adipocytes; neural stem/progenitor stem cells, which are multipotent and can differentiate into neurons, astrocytes, and oligodendrocytes; and human induced pluripotent stem cells (hiPSCs), which are adult cells taken back to a pluripotent state (Mothe and Tator, 2012). Both hESCs and hiPSCs are pluripotent, meaning they can differentiate into any cell type in the body (Itskovitz-Eldor et al., 2000). However, hESCs pose the risk of immune rejection after transplantation and remain ethically controversial because the blastocyst from which they are isolated does not survive the derivation process (Bobbert, 2006). hiPSCs are adult somatic cells reprogrammed into a pluripotent state using transcription factors (Takahashi et al., 2007). They offer the opportunity to replace cells lost while minimizing the risk of immune rejection as these cells lines can be derived directly from a patient’s own cells (Kamao et al., 2014). Neurodegenerative diseases can be modeled using hiPSCs by reprogramming adult cells taken from patients into neural cells, which then display disease hallmarks (Durnaoglu et al., 2011).

Any cell line chosen for bioprinting must have the ability to expand to sufficient numbers to be printable (Murphy and Atala, 2014). Many primary cell types cannot self-renew while being difficult to isolate, making pluripotent stem cells a more attractive option when bioprinting (Murphy and Atala, 2014). Recent advancements such as clustered regularly interspaced short palindromic repeats (CRISPER/Cas9) make it possible to correct gene mutations found in cell lines, enhancing the potential of hiPSCs for use in cell replacement therapies for treatment of neurodegenerative disease (McMahon et al., 2012). Scaffold-based strategies provide an attractive approach for culturing, expanding, and delivering cells because they offer structural support for growing cells and axons and can be loaded with chemical factors to encourage differentiation and integration with existing cell culture. 3-D bioprinting can control the spatial distribution of these factors to control cell differentiation. Biomaterial scaffolds that have supported neural cell scaffolds culture *in vitro* in mouse and rat trials include polyethylene glycol (PEG) (Freudenberg et al., 2008), modified peptide gels such as RADA16-YIGSR (Cui et al., 2016), hyaluronan (Gardin et al., 2011), fibrin (Gardin et al., 2011), and alginate (Perez et al., 2016). Many studies use extracellular matrix molecules to provide structural support such as collagen, fibrin, fibronectin, and laminin (Itosaka et al., 2009; Tate et al., 2009; Johnson et al., 2010; Elias and Spector, 2012; Lu et al., 2012; Wilems et al., 2015) and polymers such as poly(lactic-co-glycolic acid), *N*-(2-Hydroxypropyl) methacrylamide, and poly(a-hydroxy-acids) (Sykova et al., 2006).

In addition to cell therapy applications, 3-D bioprinted neural tissues can be used to model diseases and for drug discovery. Several groups have grown functional neural tissue in small tissue constructs, but these methods require long and labor-intensive culture protocols (Hopkins et al., 2015). Often the function of the resulting tissues is not fully developed, lacking fully mature neural cells and their associated function as assessed by electrophysiology (Hopkins et al., 2015). Bioprinting could create accurate, reproducible tissue constructs in a high-throughput manner, allowing for large sample sizes for evaluating electrophysiological function over time.

Cell therapy can repair damaged tissues by supplying growth factors to the injury site (Kim, 2004). To produce brain tissue constructs for drug screening, or disease modeling, the current bioprinting technologies must be changed to incorporate nutrient flow throughout the cell construct. Replacing brain tissue remains a futuristic goal, but finding a way to accurately produce neural tissue that mimics the mechanical and biochemical conditions found *in vivo*. These properties include reproducing the calcium and potassium gated voltage response for neuronal signaling (Kohler et al., 1996), displaying an elastic modulus of less than 1,000 Pa, similar to brain tissue (Georges et al., 2006), and supporting a mixed cell population to better represent the native population of neuronal and support cells. Such properties must be achieved without inducing inflammation or unexpected cellular responses. Engineering biologically accurate neural tissue requires a platform with complex controls with regards to sterilization and culture conditions as well as cell and scaffold placement.

## Culturing Neural Cells *In Vitro*

### 2-Dimensional Cell Culture

2-D culture platforms are effective in inducing early neuronal developmental structures (such as neural rosettes) from hESCs and hiPSCs, but they impose unnatural geometric constraints on the cells (Shao et al., 2015). Deriving neuroepithelial cells from hESCs and hiPSCs requires a lengthy differentiation protocol. The most common method requires the formation of embryonic bodies (EBs) followed by manual isolation of neural rosettes or adherent differentiation in combination with small molecule inhibitors that promote differentiation (Chambers et al., 2009). This process takes 17–19 days and requires several replating steps (Chambers et al., 2009). Similar conversion rates can be obtained in approximately 6 days by culturing human pluripotent stem cells on laminin coated plates in the presence of E6 media (Lippmann et al., 2014). NSCs are cultured in a similar manner either as adherent or suspension cultures but face the same geometric and morphological constraints as hESCs and hiPSCs. 2-D cultures do not exhibit the same morphology as neurons in the body because they cannot grow in 3-D. Thus, many researchers have transitioned into culturing cell lines in 3-D systems.

### 3-Dimensional Cell Culture of Neural Cells Using Biomaterials

3-D cell culture requires suspending cells within a permeable scaffold matrix, resulting in a more physiologically relevant cell microenvironment (Shao et al., 2015). NPCs derived from hiPSCs cultured in 3-D produce more neuronal cells and less astrocytes compared with cells cultured in 2-D (Edgar et al., 2017). The 3-D structure of EBs in a scaffold allows intricate cell to cell and cell to scaffold interactions not possible in 2-D culture, enabling patterned and structured cell differentiation and morphogenesis (Shao et al., 2015). Neural differentiation of stem cells has been evaluated in a number of biomaterial scaffolds, including fibrin (Robinson et al., 2015), laminin (Edgar et al., 2017), alginate (Gu et al., 2016), and PEG (Schwartz et al., 2015).

Fibrin scaffolds promote neural adhesion, proliferation, and differentiation likely because low-concentration fibrin gels possess biochemical and mechanical cues similar to those of soft tissue (Willerth et al., 2006, 2007a,b, 2008; Kolehmainen and Willerth, 2012; Montgomery et al., 2015; Robinson et al., 2015). Fibrin polymerizes under mild conditions with the addition of thrombin, but this slow process is unsuitable for extrusion bioprinting. Thus, it is often mixed with polysaccharides, such as alginate, to produce a printable bioink (Gu et al., 2016). Alginate, one of the most widely employed bioinks, polymerizes quickly with the addition of a divalent cation (Skardal and Atala, 2015). Other polysaccharides, such as gellan gum, have similar rates of polymerization (Lozano et al., 2015). However, these polysaccharides are mostly inert, resulting in limited cell adhesion (Skardal and Atala, 2015).

Laminin stimulates axonal outgrowth when added to 3-D biomaterial scaffolds, likely because it plays a role in axonal guidance and cell migration in the developing CNS (Edgar et al., 2017). Fibrin functionalized with laminin elicits higher neurite outgrowth than unmodified fibrin scaffolds (Pittier et al., 2005). PEG gels functionalized with peptides and seeded with ESC-derived NPCs, endothelial cells, MSCs, and microglia/macrophage precursors showed 3-D constructs with diverse neuronal and glial populations including vascular networks (Schwartz et al., 2015). The addition of small molecules, such as retinoic acid and purmorphamine, into 3-D culture promotes more efficient differentiation, of hiPSCs into spinal motor neurons (Edgar et al., 2017). While natural hydrogels can retain the biological activities of native ECM molecules, they suffer from batch-to-batch variability and limited possibilities for biochemical modification (Caliari and Burdick, 2016). In addition, natural hydrogels pose a risk of immunogenicity and disease transfer for clinical applications (Caliari and Burdick, 2016). By contrast, synthetic hydrogels can be more amenable for biochemical functionalization, such as growth factors, ECM adhesive motifs, and specific molecules agonistic or antagonistic to cell surface receptors, biophysical modulations, including mechanical stiffness, pore size, and 3-D architecture, and mimicking key degradation characteristics. Synthetic hydrogels also have a lower risk for immunogenic reactions as their monomers are produced using chemically defined reactions (Shao et al., 2015).

In terms of comparable technology to 3-D printing, Lancaster et al. cultured brain-like organoids, mini organs that possess similar characteristics to their human counterparts, inside of Matrigel droplets using a spinning bioreactor (Lancaster et al., 2013). After 30 days, a continuous neuroepithelium had formed surrounding a fluid-filled cavity with defined brain regions similar to the cerebral cortex, choroid plexus, retina, and meninges. Achieving a nanoscale resolution to ensure directed differentiation into unique brain areas presents one of the greatest challenges when engineering tissues (Rafat et al., 2017). The organoids reached a maximum size of approximately 4 mm after 2 months in culture. They survived up to 10 months when maintained in the bioreactor. The researchers surmised that the lack of the vascular network resulted in limited size, causing cells toward the center of the mass to die due to lack of oxygen (Lancaster et al., 2013). Bioprinting can address this important limitation of organoid formation as cell placement and their associated function could be more closely controlled by specific mechanical cues from the surrounding scaffold. Large hollow structures have already been bioprinted, but being able to incorporate blood vessels into such tissues would allow for natural vascularization (Hoch et al., 2014).

Printed scaffolds display similar degradation timelines and kinetics to their unprinted counterparts. Biomaterials for neural tissue engineering must consider that they are meant to be directly implanted or mimic natural brain tissue. Any degradation products can impact the developing or existing tissues (Wang et al., 2003). The chemical kinetics surrounding the degradation of the chosen scaffold material must be well understood to ensure the materials being released are not biologically active, or are active to a very low degree. This will depend both on scaffold composition and rate of degradation. In general, neural scaffold materials degrade via hydrolysis, ion exchange or through enzymatic reactions over a period of 2–8 weeks (Wang et al., 2003). Common degradation products include salts like calcium, protein fragments or weak acids such as lactic acid (Anderson et al., 2008). All mid- and end-point degradation products must be thoroughly investigated for possible immunogenic reactions. Possible host reactions to the biomaterial include injury, blood-material interactions, inflammation, and development of a fibrous capsule to isolate the foreign material (Anderson and Jones, 2007).

## Early Bioprinting

Bioprinting enables significant control over the arrangement of cells and bioactive nanomaterials in defined-scaffold geometries in comparison with other tissue-engineering techniques (O’Brien et al., 2015). Printing cell scaffolds means more effective composition with less effort, achieving biomimetic constructs with ECM feature size and composition, chemical gradients, varied mechanical properties, and specific morphologies that were not previously accessible (Chia and Wu, 2015). 3-D printing has been widely investigated for industrial rapid prototyping and additive manufacturing protocols (Gross et al., 2014). 3-D printing neural tissue requires creating a computer-aided design (CAD) model of the desired tissue structure including cell type and elastic moduli, input your starting materials, and letting the program associated with the 3-D printer run. The program parses the solid object into a stack of cross-sections and then prints the desired structure upwards from the bottom along the *Z*-axis (O’Brien et al., 2015).

Fabricating tissues in a controlled environment outside of a living organism requires reproducing the chemical, mechanical, and morphological properties found *in vivo* (Ahmad and Makoto, 2017). Several key components when bioprinting must be optimized to achieve *in vivo* mimicry, including the most important component—the bioink. Many natural polymers, such as fibrin, laminin, gelatin, and collagen, can be crosslinked under mild conditions into a cytocompatible hydrogel scaffold suitable for 3-D bioprinting (O’Brien et al., 2015). Many synthetic scaffold materials require complex reactions for functionalization, which hinders their ability to be bioprinted (Carrow et al., 2015). Mechanical restrictions also influence the choice of bioink when 3-D printing. Inkjet and laser-based bioprinting methods require a low-viscosity liquid, while extrusion printing requires a higher viscosity, indicating that different formulations are necessary depending on the printing method (Ahmad and Makoto, 2017). Supplements such as alginate are often added to the bioink to improve gelation speed and mechanical strength and maintain a good printing environment (Ahmad and Makoto, 2017). Another important consideration for bioink preparation is the printability, which depends on several rheological factors, including viscosity, surface tension, and thixotropy (O’Brien et al., 2015). Bioprinting requires the ability to eject the bioink, deposit, and solidify the bioink while retaining spatial resolution of the material to control and generate desired high-quality 3-D construct with accurate geometry. Thus, bioink viscosity plays a vital role in determining the flexibility of freestanding constructs and preserving their structural integrity during and after the printing process. Cells and biomolecules experience shear stress, local rheologic forces, or other external physical forces during printing process, which influences cell response (O’Brien et al., 2015). Thus, understanding how the parameters of bioprinting affect cellular processes throughout the printing process ensures the ability to obtain a viable construct (Ahmad and Makoto, 2017). Physiochemical properties (such as viscosity, elastic moduli, yield strength, reactivity, and degradation products) and cytocompatibility for the chosen cell line for printing serve as the two most important factors when designing a bioink (Ahmad and Makoto, 2017). Neuronal lineage cells derived from any source tend to be delicate and easily disrupted, presenting a major challenge when bioprinting (Potter and DeMarse, 2001). Controlling neural cell differentiation often uses defined culture conditions to ensure lineage (Ahmad and Makoto, 2017). The cell scaffold introduces a new set of proteins and biomolecules which cells will encounter during growth. The scaffold presents a 3-D microenvironment for controlling cell behavior through biophysical and biochemical cues (Ahmad and Makoto, 2017).

**Figure 1:**
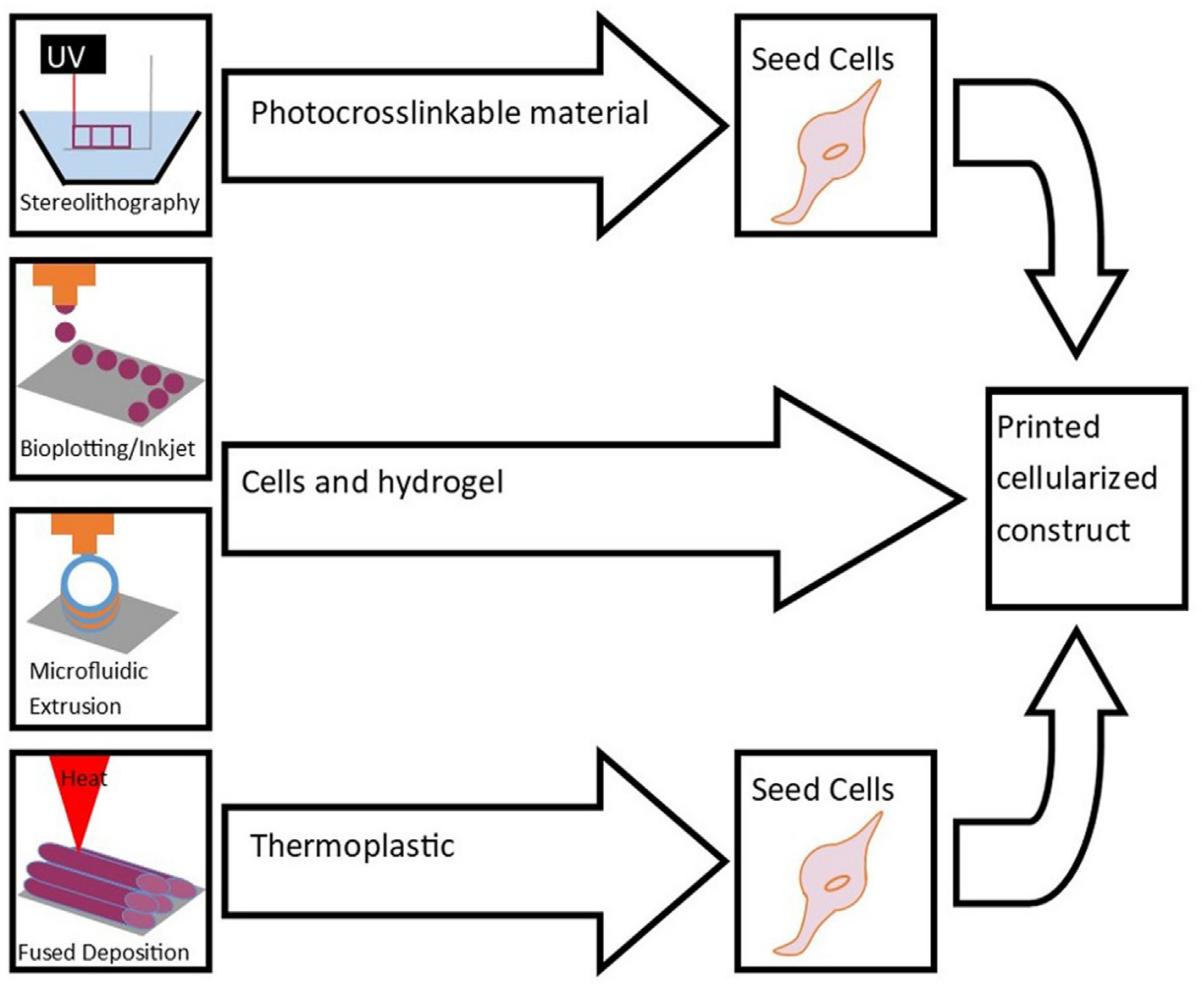
Bioprinting methods include stereolithography, bioplotting, inkjet printing, microfluidic extrusion, and fused-deposition modeling. These techniques are used to print scaffolds for cell seeding and culture to engineer tissue.

The following sections introduce several methods of bioprinting (Figure 1). These printing technologies can be improved by developing more sophisticated nozzles, cartridges that allow for automated loading, and speed and accuracy of the printing process. High resolution cell distribution remains an issue despite being improved in the last decade (Ahmad and Makoto, 2017).

### Fused-Deposition Modeling

Fused-deposition modeling (FDS) uses a melted thermoplastic which is deposited layer-by-layer onto a flat substrate to build a 3-D construct (O’Brien et al., 2015). While FDS is extremely inexpensive, it has a low accuracy (±127 μm) and height resolution (50–762 µm). The thermoplastic cannot support itself immediately when deposited, limiting potential geometries. Cells can either be encapsulated in the material prior to extruding or seeded on top of the construct. Most FDS trials have been with cells seeded on top of the scaffold for musculoskeletal applications (since the materials are harder and more compatible with bone or dense muscle tissue), but some success has been had with encapsulated cells for neural tissue engineering (O’Brien et al., 2015).

### Selective Laser Sintering (SLS)

Selective laser sintering uses a similar process as FDM, but SLS has a higher resolution (O’Brien et al., 2015). A long wavelength laser fuses beads of premade material together one layer at a time. Common materials include polycaprolactone (PCL) (Tan et al., 2005; Partee et al., 2006), polyvinyl alcohol (Chua et al., 2004; Tan et al., 2005), hydroxyapatite (Chua et al., 2004; Tan et al., 2005), and poly(l-lactic acid) (Tan et al., 2005). A layer of powder or beads is deposited, heated, and fused and then another layer deposited building up a 3-D construct. This process is both costly and slow with limited ability to remove non-sintered material. Very few materials are compatible with SLS and biocompatible. SLS, such as FDS, has largely generated scaffolds for bone tissue or other support structures for tissues (O’Brien et al., 2015).

### Stereolithography

Stereolithography is the highest resolution option for bioprinting (O’Brien et al., 2015). It can print light-sensitive polymeric materials, which often polymerize to soft substrate materials with similar mechanical cues to that of neural tissue, which helps differentiate seeded cells into neuronal subtypes (Edgar et al., 2017). In stereolithography a laser and directed mirror array project patterned light onto the surface of a resin-containing vat, curing the resin. A fresh layer of resin is added with the process being repeated to generate the desired structure. Uncured resin remains liquid, making for easy removal. This process can be used to incorporate nanomaterials, as well as growth factors and other additives without additional processes if they are not light-sensitive. Commercial systems for stereolithography use propriety nonbiomimetic inks, and the printing process can take long periods of time for printing (O’Brien et al., 2015). Stereolithography remains an understudied area with respect to applications in neural tissue engineering.

### Inkjet Bioprinting

Inkjet bioprinting uses a modified inkjet printer to deposit cells encapsulated in a bioink onto a chosen substrate (O’Brien et al., 2015). The bioink cannot have a high viscosity, often resulting in constructs with poor mechanical properties. In addition, the small nozzle size damages the cells being printed as they become deformed when passing through the nozzle. The nozzle size and flow rate also restrict the volume deposited per drop (<10 pL), meaning high concentrations of cells (greater than 5 million cells/mL) must be seeded to maximize the possibility that each drop of bioink contains one cell. However, inkjet bioprinting offers a simple process to print multiple cell types, making it useful for printing thin tissue constructs like brain slices (O’Brien et al., 2015).

### Bioplotting

Bioplotting using syringes to print tubes or spheroids layered on top of each other (O’Brien et al., 2015). Radiation, chemical reaction, or solidification then cures the material after printing. Bioprinting requires viscous bioinks as they need to hold their shape after extrusion from the needle. These bioinks tend to either be too hard or possess a low elastic modulus unsuitable for neural tissue-engineering applications. Several syringes can be used over the same substrate when placing different cell types in a desired format, but resolution is lower than microfluidic extrusion. It can print cocultured scaffolds and tissue constructions (O’Brien et al., 2015).

### Microfluidic Extrusion

Microfluidic extrusion represents an extension of bioplotting (Pfister et al., 2004). This process continuously extrudes a cell-seeded bioink-precursor in tandem with a crosslinking agent. The mixture meets in a chamber, before being extruded at the desired flow rate. The mixing initiates polymerization before deposition, allowing for easy flow through the nozzle and a defined structure after printing. Multiple valves and chambers can control of the cell type and mechanical properties of the construct. The computer-guided deposition process is hands off, allowing for aseptic conditions during printing. This method requires hydrogel precursors that polymerize into semisolid hydrogels (O’Brien et al., 2015).

## Bioprinting Neural Tissue

Several groups have bioprinted neural tissue using various cell types with varying levels of success (Table 1). In 2006, Xu et al. inkjet printed primary embryonic hippocampal and cortical neurons suspended in phosphate-buffered saline onto collagen-based biopaper (Xu et al., 2006). Circular single-layer constructs were printed and maintained in Dulbecco’s Modified Eagle Media with 10% fetal bovine serum and 5% retinoic acid. After 8 days, cell viability was 74.2 ± 6.3%, and after 15 days, cells stained positive for the neuronal marker MAP2. Electrophysiological measurements at 15 days indicated neurons had developed voltage-gated potassium and sodium channels. The same study alternating printing a layer of cells with a layer of fibrin hydrogels (Xu et al., 2006). Initially, fibrinogen was printed in a thin layer and then thrombin was printed on top. The addition of thrombin polymerized the scaffold. A single layer or neurons was then printed on top using direct cell printing. Constructs were printed 50–70 µm thick resulting in a 3-D neural sheet 25 mm × 5 mm × 1 mm. The resulting samples stained positive for DAPI, and the cells spread and exhibited neurite outgrowth after 12 days in culture.

**Table 1:** Bioprinting neural tissue by various printing methods using different cell types and bioinks.

Bioink	Cell type	Cell source	Printing method	*In vivo/in vitro*	Outcome	Reference
Cell suspension in DPBS printed on collagen biopaper	Primary embryonic hippocampal and cortical neurons	Day-18 fetal tissue from pregnant Sprague-Dawley rats	Inkjet bioprinting of NT2 cells	*In vitro*	Immunostaining and whole-cell patch clamp showed healthy neuronal phenotypes with electrophysiological activity	Xu et al., 2006
Fibrin hydrogel	Primary embryonic hippocampal and cortical neurons	Day-18 fetal tissue from pregnant Sprague-Dawley rats	Inkjet bioprinting alternating layers of fibrin hydrogel and NT2 cells	*In vitro*	Cells stained positive for DAPI and spread over the fibrin. Some cells exhibited neurite growth	Xu et al., 2006
Hyaluronic acid hydrogels grafted with laminin	Schwann cells seeded on surface	Day 15 embryonic rats	Photopatterned layer by layer	*In vitro*	Cells retained viability for 36 h, but did not adhere to scaffolds without laminin	Suri et al., 2011
Puramatrix/agarose	Dorsal root ganglia	E-15 rat pups	Digital micromirror device to crosslink polyethylene glycol, then cell material injected into the voids	*In vitro*	Cell migration and neurite extension limited to cell permissive regions	Curley et al., 2011
Polycaprolactone (PCL) microfibers and PCL with gelatin	Neural stem cells	Mouse NSC line C17.2	Stereolithography and electrospinning	*In vitro*	Fibers improved cell adhesion, aligned fibers enhanced cell proliferation, increased neurite length and directed neurite extension of primary cortical neurons along the fiber	Lee et al., 2017
Alginate, carboxymethyl chitosan, and agarose	Cortical neural stem cells encapsulated in the scaffold	Human	Microextrusion bioprinting	*In vitro*	Proliferated for 10 days with spontaneous activity and a bicuculline-induced increase calcium response, predominantly expressing gamma-aminobutyric acid	Gu et al., 2016
Polyurethane	Neural stem cells encapsulated in scaffold	Adult mouse brain	Fused-deposition manufacturing	*In vitro*	Remained viable and stained positive for β-tubulin (neuronal marker) at 7 days	Hsieh et al., 2015
				*In vivo* (zebrafish)	Implanted scaffold improved in-chorion coiling contraction (motor function) and hatching rate [central nervous system (CNS) function] in embryonic CNS-deficit zebrafish, and improved motor function and survival rate in adult zebrafish with induced TBI	Hsieh et al., 2015
Suspension in B27 Neurobasal-A medium	Retinal ganglion cells (RGCs) and glia encapsulated in scaffold	Adult male Sprague-Dawley rats	Piezoelectric inkjet printer	*In vitro*	No significant difference in survival and neurite outgrowth between printed RGCs and glia and plated cells	Lorber et al., 2014
Media with brain derived neurotrophic factor and ciliary neurotrophic factor	RGCs	Postnatal Sprague-Dawley rats	Inkjet printing onto electrospun scaffolds	*In vitro*	RGCs maintained survival and normal electrophysiological function, and displayed radial axon outgrowth	Kador et al., 2016
Collagen and fibrin, fibrin loaded with VEGF	Neural stem cells	Mouse NSC line C17.2	Microfluidic pneumatic based bioprinting	*In vitro*	Greater than 90% cell viability was observed with cells migrating toward the fibrin	Lee et al., 2010
Gellan gum modified with RGD peptide	Primary neural stem cells encapsulated in the scaffold	E18 embryos of BALB/cArcAusb mice	Handheld microfluidic device	*In vitro*	Cells remained viable at 5 days, forming neuronal networks with glial cells	Lozano et al., 2015
GelMA and PEGDA in PBS with a photo initiator and low-level light therapy	Neural stem cells seeded on top of scaffold	Mouse	Stereolithography	*In vitro*	Light stimulation promoted NSC neuronal differentiation and inhibited generation of glial cells	Zhu et al., 2017

In 2014, Lorber et al. inkjet printed retinal glial cells and disassociated retinal cells, resulting in 57% cell death in glial cells and 33% cell death in retinal cells compared with controls of unprinted cells grown on tissue culture plates (Lorber et al., 2014). No differences in neurite outgrowth or survival were observed after 5 days compared with control cultures. The high levels of cell death suggest the need for optimization of nozzle technology to reduce cell stress and deformation to improve viability post-printing.

**Figure 2:**
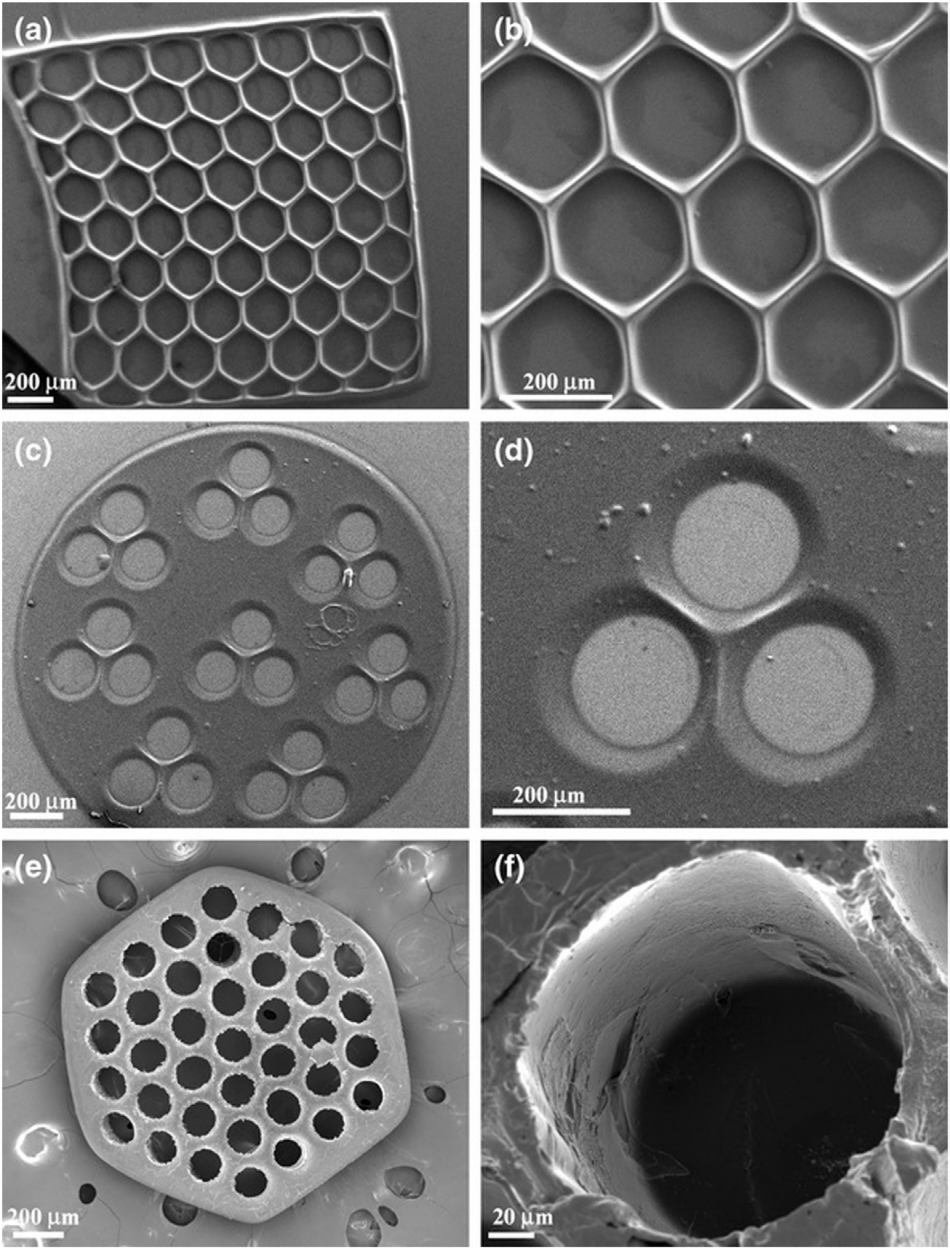
SEM micrographs of single-layered scaffolds made up of photopatterned glycidyl methacrylate and hyaluronic acid with intricate pore geometries, **(A,B)** hexagonal patterns, **(C,D)** circular patterns with three channels, and **(E,F)** circular patterns with more than 30 channels created using a digital micromirror fabrication system. Reprinted with permission from Suri et al. (2011).

Suri et al. (2011) photopatterned glycidyl methacrylate modified hyaluronic acid containing laminin using a digital micromirror device before seeding Schwann cells upon the resulting construct. Scaffolds were printed in various geometries including circles, hexagons, and squares with different pore characteristics (Figure 2). Adhered cells maintained viability after 36 h. The researchers also showed this method could be used to create gradients of fluorescent microparticles as a model for growth factor gradients, which have been shown to guide developing neurites.

**Figure 3:**
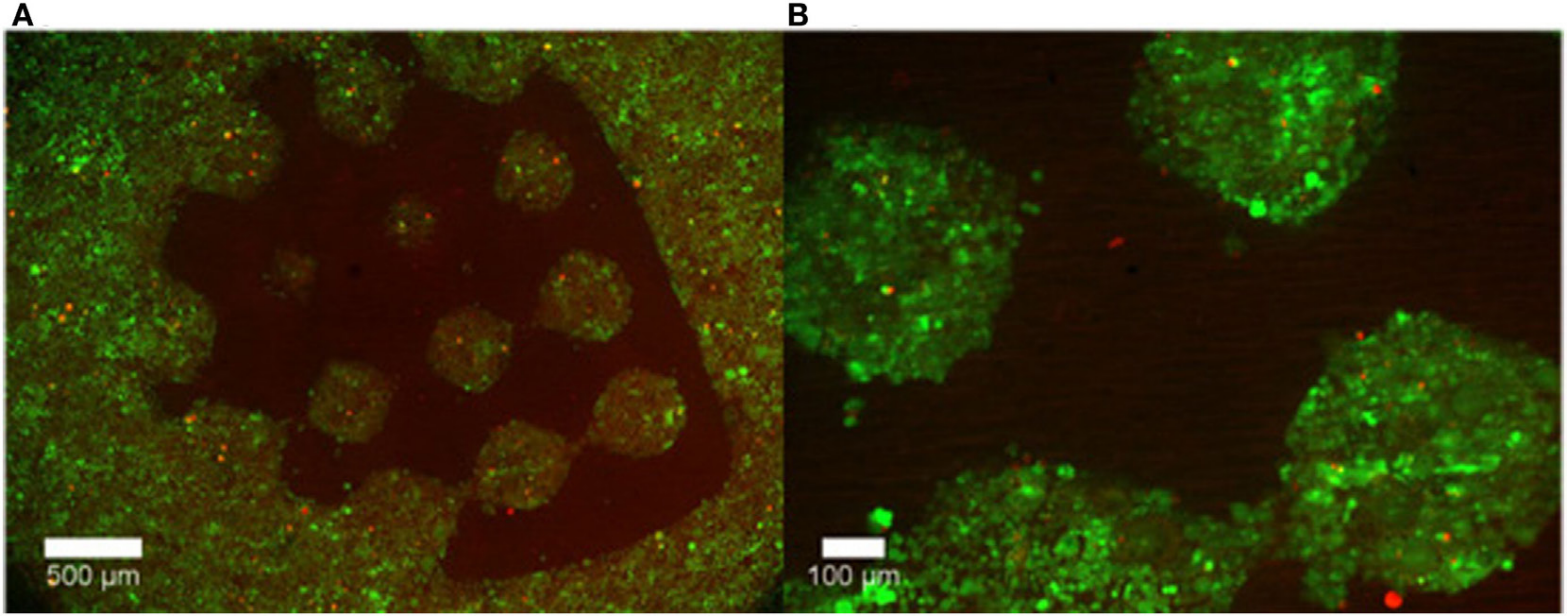
Representative images of cell growth in **(A)** the permissive region (puramatrix/agarose) versus **(B)** PEG after 48 hours. Live cells are labelled with calcein (green) while dead cells are labeled with ethidium homodimer-1 (red). Reprinted from Curley et al. (2011) under a Creative Commons License 3.0.

Curley et al. (2011) also used a micromirror array to polymerize PEG into various geometries. The voids in the PEG gel were then filled with a puramatrix/agarose cell suspension. It was shown that cells retained their viability and grew only in the cell permissive (puramatrix or agarose) region of the scaffold (Figure 3).

Lee et al. (2017) combined stereolithography and electrospinning techniques to create PCL microfibers. Scaffolds with fibers improved neural stem cell adhesion, increased neurite length, and directed neurite extensions along the length of the fiber. Zhu et al. (2017) used stereolithography to cure GelMA and PEGDA and then seeded NSCs on top of the scaffold. These constructs showed comparable viability to plated cells. Low-level light stimulation increased cell proliferation and expression of the neural marker TUJ1 (Zhu et al., 2017).

**Figure 4:**
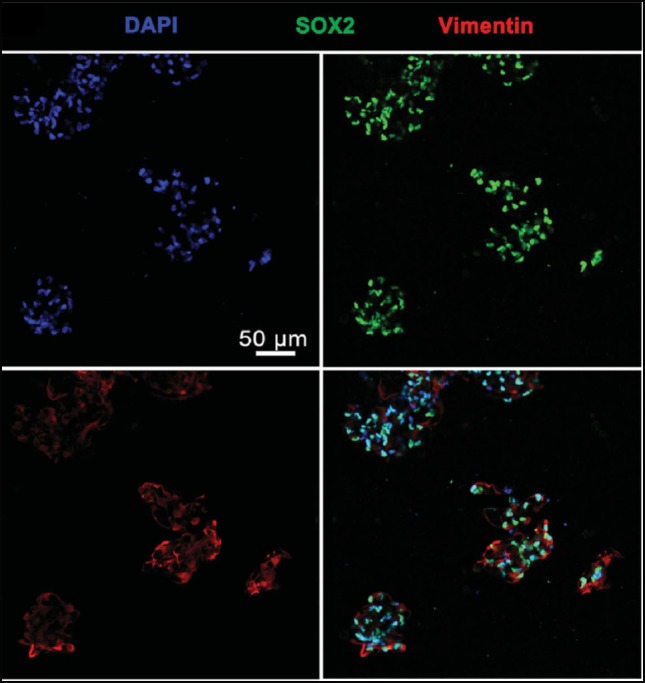
Cells Cells stained with DAPI, vimentin, and SOX2 24 days after printing. Cells largely expressed both DAPI and vimentin, indicating mature neurons. Reprinted with permission from Gu et al. (2016).

Gu et al. (2016) extruded a bioink made up of alginate, carboxymethyl chitosan (CMC) and agarose seeded with frontal cortical human NSCs. The CMC concentration influenced the cell viability. Immediately after printing 25% of seeded cells died, and cell proliferation peaked on day 11. After 3 weeks, samples stained positive for DAPI and vimentin, but had little SOX2 expression, indicating mature neurons (Figure 4).

**Figure 5:**
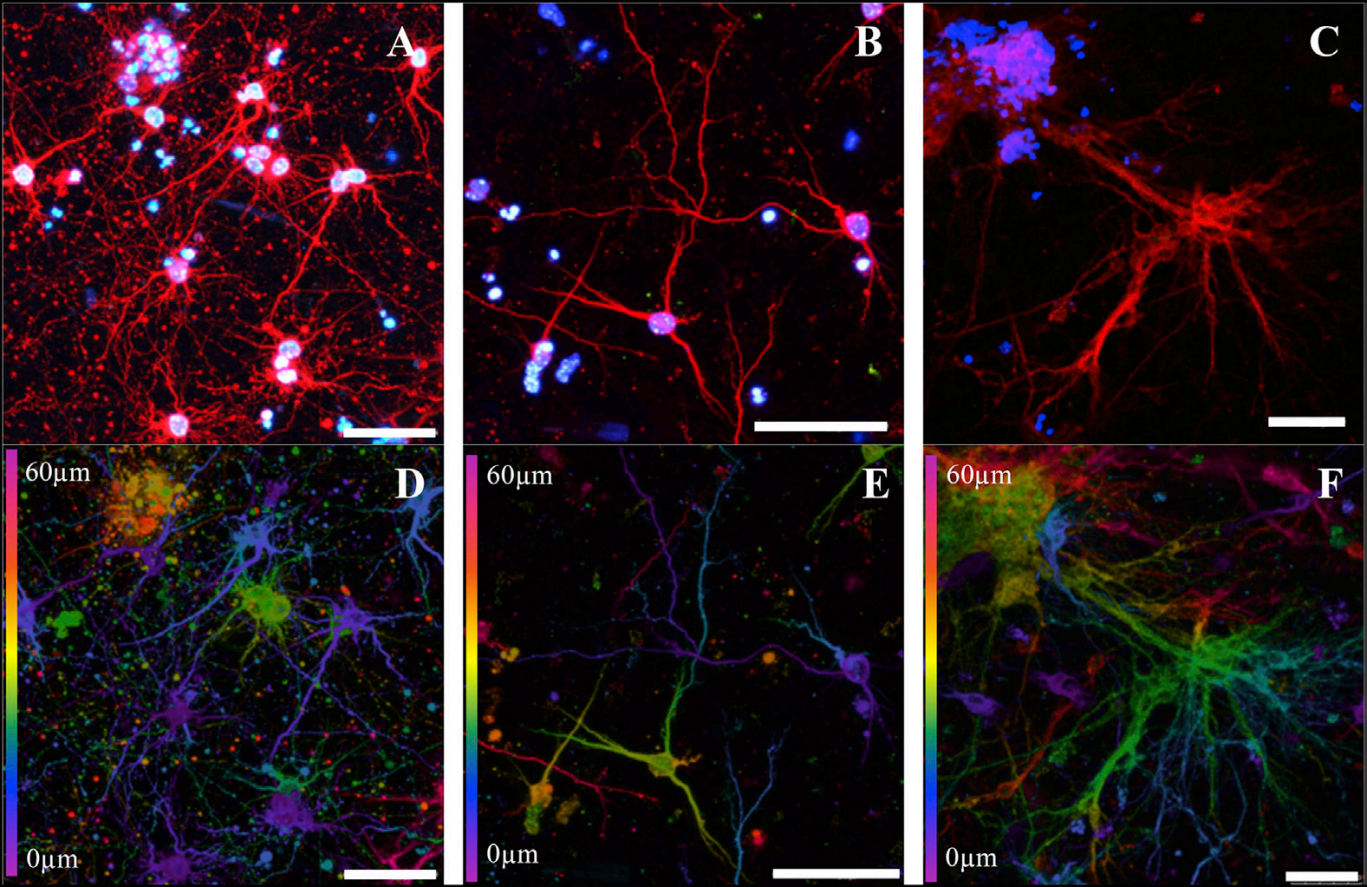
Cortical neurons encapsulated in a peptide modified gellan gum at different gel concentrations (0.075, 0.15, and 0.5% w/v, respectively) after 5 days of culture. **(A–C)** Cells stained with β-III tubulin (red) for cortical neurons and DAPI (blue) for nuclei. **(D–F)** Confocal microscope images (depth decoding) of neuronal 3-D culture models after 5 days of culture. Color decoding for the depth of the cells in the RGD-GG gel along the *Z*-axis is given (0–60 µm). Different colors represent the different planes along the *Z*-axis as shown on the sides of the images. Scale bars represent 50 µm. Reprinted with permission from Lozano et al. (2015).

Similarly, Lozano et al. (2015) extruded a peptide modified gellan gum seeded with primary cortical neurons. Cells remained viable and exhibited neuronal cell morphology after 5 days of culture and stained positive for the neuronal marker TUJ1 (Figure 5). A comparable study using FDM to print polyurethane seeded with murine NSCs by Hsieh et al. (2015) observed cell proliferation 72 h after printing. After 3 days, printed NSCs expressed more neurotrophic factor genes than NSCs cultured on tissue culture plates. The corresponding *in vivo* study implanted 3-D printed constructs into cerebellum-lesioned zebrafish. Treated fish showed increased spontaneous coiling contraction and increased hatching rate compared with lesioned untreated fish, indicating cellular restoration.

Lee et al. (2010) used microextrusion to print collagen and fibrin as well as fibrin loaded with VEGF seeded with murine neural stem cells. Constructs were printed layer-by-layer into a cylindrical shape on a tissue culture dish. Printed cells showed no difference in viability compared with manually plating cells. Cells located up to 1 mm from the fibrin border migrated toward the VEGF-containing fibrin gel, indicating that cells will migrate toward a more permissive region.

These studies differ greatly in the number of cells lost due to the stress of the printing process. Cell viability allows the user to seed at the correct cell density. However, some studies do not report cell death while others report up to 57% cell death during the printing (Lorber et al., 2014). Cell death during printing can be due to small nozzle size, polymerization or solidification reactions, or bioink composition (Zhu et al., 2017). Optimizing the bioink makeup is key to reducing the immediate loss of cell viability post-printing.

Current work indicates that a wide variety of bioink materials may be suitable for 3-D printing neural tissue. However, more research needs to be done comparing the printability of each of these materials in terms of efficiency and ease-of-use, both which become important when scaling up production. This review has covered multiple methods of 3-D printing neural constructs. Inkjet bioprinting is the most well documented but is limited in both bioink material and geometries. Microfluidic extrusion has recently seen success in printing complex shapes with various neural cell types and remains an option of interest that needs further research in creating ideal bioink compositions. Other possibilities, such as stereolithography and SLS, remain underused for neural tissue applications.

What remains to be done is finding a cohesive unit of bioink and bioprinting method which results in a high cell viability post-printing and is adaptable enough to print multiple different neural cell types with a bioink which has controllable elastic properties and porosity and can be loaded with factors to further control differentiation.

In addition, most studies lack a hands-off manner of controlling bioprinting. Incorporating CAD and microtechnology into printing projects would help fully realize the high-throughput nature of 3-D bioprinting tissue, as the field is still largely limited by human-controlled systems. The use of CAD would further assist in increasing cell resolution within printed constructs. Advancing the resolution of bioprinting could also allow the printing of vascular networks within a designed tissue, something which would allow neural models to be scaled-up beyond a maximum achieved size of mm. This development would allow more physiologically relevant constructs to be printed for disease modeling and drug discovery.

## Conclusion

Bioprinting can change how neural tissue are engineered, moving it from a time consuming, hands-on process that can vary from lab-to-lab to a sterile, high-throughput process that can rapidly produce physiologically accurate brain constructs for applications in cell therapy and drug screening. The low throughout methods for engineering brain tissue limit their applicability for drug screening. Cell therapy has had limited success for the same reason: the number of cells required for injection requires lengthy culture time in addition to the difficulty controlling cell diffusion and differentiation. For bioprinting to succeed as the new standard for engineering neural tissue more bioinks must be done to accurately control brain region development, and the issue of vascularization must be solved to print accurate constructs suitable for long-term culture. However, such bioprinted neural tissues hold great promise for applications in both cell therapy and for drug screening.

## Author Contributions

MT and SW both contributed to the authorship. SW proposed the topic and provided feedback through the writing process. MT wrote the initial draft and completed revisions based on feedback provided.

## Conflict of Interest Statement

The authors declare that the research was conducted in the absence of any commercial or financial relationships that could be construed as a potential conflict of interest.
